# Beyond Pneumonia: A Dual-Cycle Initiative to Improve Imaging Compliance and Uncover Early Malignancy

**DOI:** 10.7759/cureus.89417

**Published:** 2025-08-05

**Authors:** Manzoor Wani, Abir Aijaz, Abdul Bhat, Amit Badshah, Muhammad Hamid, Shayekh Ferdoush, Shovan Rehman, Ayesha Babar

**Affiliations:** 1 Acute Medicine, University Hospitals Bristol And Weston, Weston-super-Mare, GBR; 2 Internal Medicine, University Hospitals Bristol and Weston, Weston-super-Mare, GBR; 3 Acute Medicine, University Hospitals Bristol and Weston, Weston-super-Mare, GBR; 4 Acute and General Internal Medicine, University Hospitals Bristol and Weston, Weston-super-Mare, GBR; 5 Respiratory Medicine, University Hospitals Bristol and Weston, Weston-super-Mare, GBR

**Keywords:** ca lung, chest-x-ray (cxr), community-acquired pneumonia (cap), ct chest, quality improvement projects

## Abstract

Community-acquired pneumonia (CAP) is a significant cause of morbidity and mortality in adults. National guidelines by the British Thoracic Society (BTS) and the National Institute for Health and Care Excellence (NICE) recommend follow-up chest imaging within six weeks for adults diagnosed with CAP to exclude underlying malignancy. Timely follow-up of radiological abnormalities in CAP is crucial, as infectious infiltrates can obscure early signs of malignancy. At our institution, follow-up imaging was often delayed or missed entirely, risking delayed cancer diagnosis. This quality improvement project (QIP) sought to quantify adherence to national guidelines and to assess whether low-cost interventions could improve compliance. The implementation of targeted, low-resource interventions significantly improved compliance with national guidelines. Ongoing efforts are necessary to sustain improvement and optimise patient outcomes.

## Introduction

Community-acquired pneumonia (CAP) is among the leading causes of hospital admissions and is associated with considerable morbidity, particularly among older adults and those with comorbidities [1]. While pneumonia is often self-limiting with appropriate treatment, it can mask underlying pulmonary malignancy, particularly in older adults and smokers [2]. The British Thoracic Society (BTS) guidelines recommend that all patients aged ≥50 years, and those at higher risk, should undergo a repeat chest X-ray six weeks after diagnosis to confirm the resolution of infection and exclude malignancy [3]. The National Institute for Health and Care Excellence (NICE) guidelines endorse these recommendations [4]. Despite their clinical importance, adherence to these guidelines is often suboptimal in practice [4,5]. Failure to follow up may delay cancer diagnoses and compromise patient outcomes. This quality improvement project (QIP), conducted in two cycles, aimed to quantify local compliance with follow-up imaging guidelines and implement strategies to address the gaps in practice.

## Materials and methods

This prospective, two-cycle research project within the quality improvement domain was carried out over 16 weeks at a district general hospital in the United Kingdom. The project was initiated and led by a team of junior doctors and middle-grade doctors, with oversight and support from the Acute Medicine department. The primary aim was to assess and improve compliance with national guidelines recommending follow-up chest imaging for patients diagnosed with CAP. The specific objectives were twofold: first, to determine the proportion of patients with radiologically confirmed CAP who received follow-up imaging within six weeks of diagnosis, as per BTS and NICE guidelines; and second, to increase this proportion by a significant number through the implementation of targeted, low-cost interventions.

Inclusion and exclusion criteria

The audit included adult patients aged 18 years and above who were admitted to the Acute Medical Unit (AMU) with a new diagnosis of CAP confirmed on chest radiography during the admission. Patients were excluded if they had a known diagnosis of primary lung cancer or metastatic lung disease, or if they were discharged to palliative care, as follow-up imaging was not clinically indicated in these groups.

Cycle 1: baseline data collection

In the initial audit cycle, conducted over eight weeks, 42 eligible patients were prospectively identified. For each patient, the following data were recorded: age, sex, date of CAP diagnosis, whether follow-up imaging was arranged before discharge, whether imaging was completed within six weeks, and the modality of imaging (chest X-ray or CT scan). The baseline audit revealed that only a small number of patients received follow-up imaging within the recommended timeframe. A review of patient records and discharge documentation identified several common barriers to compliance, including a general lack of awareness among staff regarding the guidelines, failure to include imaging recommendations in discharge summaries, and the absence of formal follow-up arrangements or clinic referrals.

Intervention strategy

In response to these findings, a series of interventions was implemented over the following weeks. Educational materials summarising the audit results and BTS/NICE guidelines were circulated to both medical and nursing staff via departmental emails. Posters and banners were placed in high-traffic clinical areas, such as doctors’ offices and ward stations, to serve as visual reminders of follow-up requirements. Additionally, patient-facing leaflets were designed and included in discharge folders, encouraging patients to attend scheduled follow-up imaging. Finally, doctors were encouraged to use standardised discharge summary templates, which included a prompt to consider arranging follow-up chest imaging for patients with CAP.

Cycle 2: post-intervention re-audit

Eight weeks after the interventions were rolled out, a second audit cycle was conducted. This cycle included 50 patients who met the same inclusion criteria as in Cycle 1. The same data points were collected to assess the impact of the interventions on clinical practice and guideline adherence.

## Results

A total of 42 patients were included in the initial cycle, with 22 undergoing repeat imaging (X-ray or CT scan), while 16 did not receive follow-up imaging, and four patients died before the six-week follow-up. Among those who underwent repeat imaging, two were diagnosed with cancer.

Gender-wise distribution for repeat imaging

Out of 42 patients, 22 were male and 20 were female. Repeat imaging (Table 1) was performed in 12 of 22 (54.5%) males and 10 of 20 females (50%). A Chi-square test showed no significant association between gender and the likelihood of receiving repeat imaging (χ² = 0.17, p = 0.68).

**Table 1:** Repeat imaging: gender-wise distribution

Gender	Repeat imaging done	Repeat imaging not done	Total
Male	12	10	22
Female	10	10	20
Total	22	20	42

Cancer detection by gender

Among the 12 males who had repeat imaging, one was diagnosed with cancer (Table 2). Similarly, one out of 10 females who had repeat imaging was found to have cancer. Chi-square analysis revealed no statistically significant association between gender and cancer diagnosis (χ² = 0.02, p = 0.89).

**Table 2:** Cancer detection: gender-wise distribution

Gender	Cancer detected	Cancer not detected	Total
Male	1	11	12
Female	1	9	10
Total	2	20	22

Age group distribution for imaging

Patients were categorised into three age groups (Table 3): 20-49 years (n = 12), 50-70 years (n = 16), and over 70 years (n = 14). Repeat imaging rates across age groups were 50% in the 20-49 years group, 50% in the 50-70 years group, and 57.1 in the over 70 years group, respectively, with two people diagnosed with cancer. Chi-square testing did not demonstrate any significant difference in imaging follow-up by age (χ² = 1.18, p = 0.55) or cancer detection across age groups (χ² = 0.97, p = 0.61).

**Table 3:** Repeat imaging: age group-wise distribution

Age group, years	Repeat imaging done	Repeat imaging not done	Died before six weeks	Total
20-49	6	6	0	12
50-70	8	7	1	16
>70	8	3	3	14

Cancer detection by age group (patients with repeat imaging)

There was no statistically significant association between repeat imaging (Table 4) or cancer detection and gender or age group in this cohort. Despite follow-up imaging identifying cancer in two patients, demographic variables did not predict imaging follow-up.

**Table 4:** Cancer detection by age group in patients with repeat imaging

Age group, years	Cancer detected	Cancer not detected	Total
20-49	0	6	6
50-70	1	7	8
>70	1	7	8

Gender and repeat imaging 

Chi-square (χ²) = 0.020, p = 0.886. There was no statistically significant association between gender and the likelihood of having repeat imaging (p>0.05). The difference in follow-up imaging rates between males (n = 8, 71.4%) and females (n = 5, 77.3%) appears minimal and may be attributed to chance (Table 5).

**Table 5:** Gender and repeat imaging

Gender	Repeat imaging	No repeat imaging	Total
Male	20	8	28
Female	17	5	22
Total	37	13	50

Gender vs. cancer detection (among patients with repeat imaging, n = 37) 

Chi-square (χ²) = 1.127, p = 0.288. While three (15%) of the males who had repeat imaging were found to have cancer, none of the females were diagnosed with malignancy. However, this difference was not statistically significant (p>0.05). The observed trend may suggest a higher malignancy yield in males, but a larger sample size is needed for confirmation (Table 6).

**Table 6:** Genderwise data related to cancer detection in patients with repeat imaging

Gender	Cancer detected	Cancer not detected	Total
Male	3	17	20
Female	0	17	17
Total	3	34	37

Age group vs. cancer detection (among patients with repeat imaging, n = 37)

Chi-square (χ²) = 1.983, p = 0.371. Although a higher number of cancer cases were seen in patients >70 years (n = 2, 15.4%) compared to other age groups (1/12 in 50-70, and 0/12 in 20-49), the differences were not statistically significant (p>0.05). This pattern may indicate an age-related risk for malignancy but requires a larger cohort to achieve statistical power (Table 7).

**Table 7:** Age group-wise data related to cancer detection in patients with repeat imaging

Age group, years	Cancer detected	Cancer not detected	Total
20-49	0	12	12
50-70	1	11	12
>70	2	11	13
Total	3	34	37

The two-cycle QIP revealed that 57.89% of patients had a chest X-ray performed as per national guidelines, and patients less than 50 years old were only considered for this study if they were smokers. The proportion of people who were diagnosed with cancer after going for repeat imaging was nearly 9% in total, which is a significant number if diagnosed at this stage.

## Discussion

CAP remains a significant cause of morbidity and mortality in adults, particularly in the elderly and those with underlying comorbidities. Chest radiography plays a central role in the diagnosis and management of CAP, not only by confirming the presence of parenchymal infiltrates but also in ensuring radiographic resolution post-treatment. BTS and NICE guidelines advocate for follow-up chest imaging-typically within six weeks of the initial diagnosis in adults who have been admitted with radiologically confirmed CAP to exclude underlying malignancy or persistent infection [5,6]. The rationale for this recommendation lies in the well-documented association between pneumonia and the subsequent diagnosis of lung cancer. Studies have shown that a significant proportion of patients initially treated for CAP are later diagnosed with primary pulmonary malignancies, often within a short window following their pneumonia episode [7]. The obstructive effects of a tumour can lead to post-obstructive pneumonia, and conversely, infiltrates caused by infection may mask radiological features suggestive of malignancy. Therefore, radiographic follow-up assumes a dual role: confirming the resolution of infectious changes and serving as a safety net for detecting undiagnosed neoplastic processes [8].

Despite the clarity of national guidelines, adherence remains suboptimal in clinical practice. Multiple studies across the UK have demonstrated poor compliance with follow-up imaging, with reported follow-up rates ranging from 30% to 60% in various hospital settings [9-11]. Reasons for this include the absence of clear discharge documentation, lack of communication between inpatient and outpatient services, and limited patient awareness regarding the importance of follow-up imaging. Interventions such as improved discharge summaries, structured care bundles, and patient education materials have been proposed to bridge this gap [12]. Furthermore, it should be ensured that radiographic follow-up is not merely a matter of guideline adherence; it holds significant implications for public health and early cancer detection. Lung cancer, when detected early, is far more amenable to curative treatments, including surgical resection and stereotactic radiotherapy. A delay in diagnosis, particularly due to missed opportunities such as a failure to arrange timely imaging, can result in the disease being diagnosed at a more advanced and less treatable stage. Thus, CAP episodes in high-risk populations, including smokers and those over 50, represent a critical window for early detection of malignancy [13].

Quality improvement initiatives in this context offer a valuable strategy to identify local barriers to compliance and implement targeted interventions. A typical QIP cycle, involving baseline data collection, dissemination of findings, and implementation of changes, followed by re-audit, can effectively raise awareness among healthcare professionals and improve practice patterns. Educational materials, such as trust-wide emails, posters, and clinical leaflets, serve as low-cost but effective methods to enhance knowledge and promote best practices [14]. Additionally, system-level approaches, such as incorporating radiographic follow-up into electronic discharge templates or flagging eligible patients via hospital information systems, can automate the process and reduce reliance on individual clinician memory or initiative. Integration with primary care services is equally important. General practitioners must be aware of the recommendations and encouraged to follow up with patients who may have been discharged without a scheduled imaging appointment.

Nevertheless, certain challenges remain. The COVID-19 pandemic has further strained imaging services, resulting in delays in routine follow-up scans, including those for CAP. As services recover, prioritising high-risk patients and ensuring adherence to BTS and NICE guidelines becomes even more critical [15]. Moreover, ensuring equity in follow-up access, particularly for socially or economically disadvantaged groups, remains an area of concern that future QIPs must address. Thus, follow-up chest imaging in patients with CAP is a vital component of holistic, evidence-based care. It plays a critical role in the early detection of lung cancer and ensures complete resolution of infectious changes. Adherence to national guidelines must be continually evaluated and improved through structured QIPs. A multidisciplinary approach, involving clinicians, radiologists, discharge planners, and primary care providers, is essential to close existing practice-related gaps and improve patient outcomes.

Limitations

This study has a few limitations. We employed a single-centre design, and hence the findings may not be generalisable. Moreover, the study was conducted over a relatively short time frame between audit cycles, and reliance on accurate documentation for follow-up verification was high. Additionally, no qualitative data were gathered from staff regarding barriers to implementation.

## Conclusions

The research project proved that simple educational and administrative changes can lead to significant improvement. Involving a multidisciplinary team and empowering patients to follow up can increase compliance. It also highlighted that sustaining the change requires incorporation steps into institutional processes, such as discharge summaries and electronic health record prompts. This QIP demonstrated that compliance with BTS/NICE recommendations for follow-up imaging in CAP can be significantly improved through cost-effective, staff-led interventions. Ensuring timely follow-up is essential to exclude underlying pathologies such as lung cancer. We recommend that healthcare providers integrate follow-up prompts into discharge documentation and consider automated reminders within electronic systems. Future steps should focus on the importance of conducting quarterly re-audits to ensure sustained compliance, embedding prompts in electronic discharge summaries, and expanding to other departments, including Emergency and Respiratory Medicine.
